# COVID-19 and Essential Workers: A Narrative Review of Health Outcomes and Moral Injury

**DOI:** 10.3390/ijerph18041446

**Published:** 2021-02-04

**Authors:** Joanna Gaitens, Marian Condon, Eseosa Fernandes, Melissa McDiarmid

**Affiliations:** School of Medicine, University of Maryland, Baltimore, MD 21204, USA; jgaitens@som.umaryland.edu (J.G.); eseosa.fernandes@som.umaryland.edu (E.F.); mmcdiarm@som.umaryland.edu (M.M.)

**Keywords:** COVID-19, essential workers, moral injury, worker protections

## Abstract

The COVID-19 pandemic has introduced a number of added obstacles to safe employment for already-challenged essential workers. Essential workers not employed in the health sector generally include racially diverse, low-wage workers whose jobs require close interaction with the public and/or close proximity to their coworkers, placing them at increased risk of infection. A narrative review facilitated the analyses of health outcome data in these workers and contributing factors to illness related to limited workplace protections and a lack of organizational support. Findings suggest that this already marginalized population may also be at increased risk of “moral injury” due to specific work-related factors, such as limited personal protective equipment (PPE) and the failure of the employer, as the safety and health “duty holder,” to protect workers. Evidence suggests that ethical and, in some cases, legally required safety protections benefit not only the individual worker, but an employer’s enterprise and the larger community which can retain access to resilient, essential services.

## 1. Introduction

### Essential Workers

As the COVID-19 pandemic swept across the globe in 2020, nothing became more apparent than the social inequities that exist between different groups of workers. While many businesses began to close and numerous workers lost their jobs, those who remained working began to face a new set of challenges that often differed by social and economic class [1,2]. For example, some middle- and upper-income workers, such as those working in the technology industry, were able to transition from working in an office to working from home, whereas workers in many so-called essential industries, who often receive lower wages, were not afforded the same work-from-home opportunity [2], placing them at greater risk for exposure to COVID-19 [3,4,5,6].

The International Labor Organization (ILO) has defined essential services as services “without which the safety, health or welfare of the community or a section of the community would be endangered or seriously prejudiced” [7]. At the start of the pandemic, many countries scrambled to identify specific services and groups of workers that they considered essential [8]. While this list varies by country, and sometimes within country, workers in healthcare, food and agriculture, public utilities and safety, manufacturing, transportation, and communications often top the list [9,10]. As an example, in the U.S., the exact definition of who qualifies as an essential worker varies from state to state. However, in late March 2020, the Cybersecurity and Infrastructure Security Agency under the U.S. Department of Homeland Security issued guidance to assist state, local and other authorities in identifying the “Essential Critical Infrastructure Workforce” [11]. As shown in Table 1, this workforce includes all of the main sectors listed above and is very broad, covering approximately 70% of all U.S. workers [12]. Workers in these industries are considered vital for maintaining the health, safety, and well-being of the community in times of an emergency and therefore must continue to work and provide services during the current pandemic [11].

Although some workers in essential industries are able to work remotely, the majority must continue to report to work, placing them in close proximity to others and on the “front lines” for potentially being exposed to the virus, thereby increasing their risk for illness and death [5,6,12]. One obvious example of “front-line” essential workers are healthcare personnel. As these individuals are at especially high risk for exposure to COVID-19 due to the nature of their work caring for the sick, their health and safety at work has often been highlighted in the literature and various media reports [13,14,15,16]. However, it is equally important to recognize the impact COVID-19 has had on the health and safety of other essential “front-line” workers, such as grocery store, food processing, farm, mass transit, and public safety workers. This latter group of “front-line” workers, who tend to be low-income and more racially diverse than other workers [1,6,17,18,19,20], are often already at increased risk for experiencing ill health due to numerous social and economic disadvantages that existed prior to the pandemic [19,21,22]. For example, many essential workers employed in precarious jobs, with low pay and job security, are immigrants or migrant workers, making them more vulnerable to systemic and structural racism, which can increase their susceptibility to social and health inequities [23,24]. In addition, there is a disproportionately high number of women, another marginalized population, in some essential work sectors. For example, 71% of cashiers in grocery stores and other essential retail stores are women [4]. A recent analysis of 2018 American Community Survey data suggested that approximately 32% of grocery store workers are from low-income households (making less than $40,000 per year) and 11% of all essential workers are uninsured [19], placing these workers at greater risk if they do become ill. 

As such, this manuscript aims to describe the occupational impact of the COVID-19 pandemic on essential workers not employed in the health sector by: (1) providing examples that highlight both the physical and mental health effects; (2) describing the essential workers’ risk for “moral injury,” defined as psychological stress resulting from an act, or omission of an act from a leadership figure that betrays one’s moral or ethical code [25]; (3) identifying recurring themes of work-related, system- level failures that may increase risks to these workers; and (4) discussing key occupational health and safety elements that all employers should implement to mitigate risks. 

## 2. Methods

Given an incomplete scientific literature on this topic, as the pandemic unfolded, a narrative review was conducted to facilitate analyses of health outcome data in essential workers and work-related contributing factors to illness. Peer-reviewed, as well as gray literature and news sources, from the start of the pandemic through early December 2020, were identified using a combination of key words and phrases in search engines including PubMed and Google. These included words and phrases such as: “essential worker,” “grocery workers and COVID,” “manufacturing workers and COVID,” “meat packers and COVID,” “transportation workers and COVID,” “COVID-19 infection in workers,” “occupational risks related to COVID,” “workers at risk for COVID,” “work-related COVID infections and deaths,” “essential worker concerns during COVID,” “mental stress in essential workers,” “moral injury in essential workers” and “risk factors for moral injury during COVID.” Titles and abstracts were scanned to determine the focus of the article and only articles that discussed COVID-related illness and death data or work-related concerns due to COVID among essential workers were reviewed. Articles lacking this information were excluded from review, as were articles focused solely on workers in the health sector (as identified in Table 1). Reference lists of all articles selected for review were also scanned to identify additional articles. 

To scrutinize the evidence for essential workers’ risk for illness, details regarding the number of infections and deaths among essential workers were extracted from articles reviewed. In addition, the literature and news sources were reviewed to capture worker concerns and mental stressors related to being an essential worker during the pandemic. In total, 525 articles from the scientific literature were identified, 483 were excluded after review of the title/abstract, and 42 were reviewed.

## 3. Results

### 3.1. Evidence of Raised Infection Rates among Essential Workers

#### 3.1.1. COVID-19 Illnesses and Deaths Related to Occupation

In the U.S., there has been no systematic collection of occupational data for COVID-19 illnesses and deaths [26]. This is, in fact, a global problem. However, there have been several media reports, especially in the early months of the pandemic, as well as reports in the peer-reviewed literature describing COVID-related illnesses and death among various groups of essential workers (Table 2).

Several highly cited reports, both in the U.S. and Europe, focus on food workers with special emphasis on those in the meat packing industry [27,28,29,30,31,32,33]. These essential workers tend to work on long production lines and in close proximity to their coworkers, live in crowded conditions, and share transportation to work, thereby increasing their risk of acquiring an infection [27]. Evidence has shown that these workers tend to have high rates of infections compared to surrounding communities, forcing the closure of some plants. As an example, one Italian plant was forced to temporarily close after an estimated 25% of the workforce were reported to have fallen ill [31]. 

Grocery store employees have also been found to be at high risk for developing infection. Factors that increase their risks include encountering a high volume of customers (who may or may not wear a mask, especially in the early days of the pandemic) and the inability to social distance. One recent study found that 20% of grocery store workers tested positive for COVID-19 [36]. Although a large percentage (76%) of these workers were asymptomatic, the rates of infection were higher than rates reported in surrounding communities [36].

Emergency services personnel, such as firefighters and police, who interact with the public, are also at increased risk for COVID-19 infection. One recent study of New York City (NYC) firefighters and emergency services personnel, including medical technicians and paramedics, found that the COVID-19 infection rate in this group was 15 times higher compared to the general public during the first wave of the pandemic. Within this group, emergency services personnel had the highest rates of infections, four times that of firefighters [39].

There have also been documented reports of COVID-19 infections and deaths among correctional officers. Throughout the world, prisons have been recognized as an ideal environment for the spread of infections due to their lack of funding and overcrowding [46]. One study using state and federal U.S. prison data found COVID-19 infection rates five times higher in prisoners than the general public [47]. The potential for high rates of COVID infection in these environments, combined with the inability to social distance, limited PPE availability, and restricted use of disinfection products due to safety concerns, suggests that correctional officers and others working in prisons are at increased risk [48].

Documented infections and deaths among other types of essentials workers, including transportation and factory workers, have also raised concerns about transmission of the virus in the workplace [38,42,43,44]. Further suggesting that a person’s work matters, studies have shown increased rates of COVID-19 infection in neighborhoods that have a high percentage of essential workers [49]. One study examining data from the early months of the pandemic found that the presence of a livestock processing or meat packing plant in a county increased rates of COVID-19 infection by 51–75% and death by 37–50% above baseline [50]. According to the researchers, this finding also suggests that workplace exposures can significantly accelerate the spread of infection within surrounding communities.

While reports such as these raise concern and provide some evidence that occupation poses a risk, the numbers of those affected are likely only the tip of the iceberg as employees may not undergo systematic testing and employers may not be adequately capturing and reporting information on the number of positive cases in their workforce. 

#### 3.1.2. Complaints in the Workplace

Essential workers may not feel comfortable approaching their employer if they have concerns in the workplace as they may fear retaliation and potential job loss [51]. However, in the U.S., workers who feel that there is a significant health or safety risk in their workplace have the right to file a confidential complaint to the Occupational Safety and Health Administration (OSHA), which can trigger a workplace investigation. Interestingly, a recent review of OSHA complaint data showed that the distribution of complaints since early March 2020 mirrored the epidemic curve. The data also showed a strong correlation between the number of complaints filed and COVID-related deaths that occurred 16 days later [26]. As suggested by Hanage and colleagues, these findings may indicate that the concerns expressed by workers regarding potential health and safety violations in their workplace are real and proper investigation into these concerns can provide an opportunity for intervention [26]. However, enforcement data show that only a small percentage (2 to 4%) of worker complaints related to COVID-19 have been investigated at either the state or federal OSHA level [26]. 

#### 3.1.3. The Essential Worker and Moral Injury

Many “essential” workers are in a “no win” situation, forced to choose between the benefits of continued employment and the potential for increased risk of illness and death for themselves, their family, and their community. For these workers, who may not have even thought of themselves as being “essential” before [52], the decision they must make can be overwhelming and adds to their already significant stress. The psychological impact of COVID-19 has also been reported to be significantly higher among grocery store employees than the general population [53]. Their worries of becoming infected while at work and of infecting a loved one were even higher than those reported by healthcare workers [53].

Also, of note, workers who interacted with customers were five times more likely to test positive and those who were unable to “socially distance” reported significantly higher rates of anxiety and depression compared to other grocery store workers [36]. Studies like this suggest that essential “front-line” workers are at risk for experiencing significant mental health issues related to the stress of working during a pandemic. 

As many essential workers are low-income wage earners, they may feel as though they have no choice except to work even when conditions are unsafe, as the prospect of not receiving a paycheck can have significant economic and health consequences for themselves and their families. This may create feelings of despair and outrage toward their employer and their personal situation. In addition, the fear of bringing the virus home can increase psychological distress among essential workers [53]. Evidence showing increased rates of infection in communities with high numbers of essential workers suggests that this fear is warranted [49,50]. If a family or community member becomes ill because a worker goes to work and brings the virus home, it may result in an overwhelming sense of grief or guilt for that worker. Thus, these workers must rely on their employers to protect their health and safety, even as they enter the workplace with no such guarantees. When an employer fails to do so, these essential workers may also experience “moral injury” as well as a COVID exposure threat.

Moral injury is defined as the act, or omission of an act, from a leadership figure that betrays one’s moral or ethical code that results in profound psychological stress [25]. This concept was first used to describe the effects of perceived injustices military service members faced when placed in a position by their leadership that they felt was immoral. As described by Shay, for moral injury to occur, there must be a high-stakes situation in which a person of authority conducts themselves in a manner which betrays a person’s obligation to do what is morally right [54]. The harm that occurs often arises from trusting authorities to do the right thing when they do not. Affected individuals can experience significant grief, guilt, remorse, shame, despair, or outrage [55]. They may also lose trust in themselves and feel that others will judge them for their actions. Such circumstances can have long-lasting emotional, social, and psychological effects on affected individuals [56]. 

### 3.2. Work-Related Factors that Can Increase Moral Injury Risk in “Front-Line” Workers

The emphasis on keeping critical infrastructure industries operational during times of a crisis requires balancing the protections of individual workers against ensuring that the overall needs of the community are met [11]. This can result in limited protections for essential workers. A number of political, social, and economic factors can result in unfavorable working conditions and limited protections for working populations. Related to these, displayed in Table 3 and described below are specific workplace-level factors that may increase the essential workers’ risk of moral injury during the COVID-19 pandemic.

#### 3.2.1. Lack of Specific Federal Regulations or Standards

In the U.S., there currently is no federal OSHA standard or regulation that specifically outlines the precautions that employers are required to implement to control COVID-19 exposure in the workplace. However, the OSHA “General Duty Clause” states that employers have the obligation to provide an environment free from recognized hazards that can cause or are likely to cause death or serious harm to its employees [57]. This “duty of care” requires employers to comply with a certain standard of safety practice. In the case of COVID-19, OSHA’s General Duty Clause would obligate the employer to follow recognized standards of safety and health practices such as those from the U.S. Centers for Disease Control and Prevention (CDC) and National Institutes for Occupational Safety and Health (NIOSH) recommendations, to control exposure and disease transmission in the workplace. Such general expectations of employers as “duty holders” toward a worker’s health and safety is seen more broadly across many industrialized countries [58]. 

The recommendations put forth by such agencies may be industry specific, raising concerns that increased risks may be “more acceptable” within certain working populations. For example, in order to keep critical industries open, CDC published guidance on April 8, 2020, stating that critical infrastructure workers potentially exposed to COVID-19 can continue to work without quarantine, as long as they remain asymptomatic and take additional precautions such as wearing a mask at all times in the workplace [59]. This directive conflicted with CDC’s guidance for the general population at that time, which advised anyone exposed to COVID-19 to self-isolate by staying home and keeping at least six feet from others for 14 days [60]. As there is evidence of disease transmission from asymptomatic individuals, infected essential workers who are told they must work in the absence of symptoms may feel responsible if a coworker contracts COVID-19, thus increasing the individuals’ risk of “moral injury.” 

Employers who do not follow recommendations provided by the federal agencies may be fined under the “General Duty Clause.” However, the guidance provided to employers permits feasibility to be considered when adopting protective measures, thus leaving open to interpretation what can be implemented in certain workplace settings. Also, without proper enforcement, there is an increasing reliance on employers’ voluntary adherence to the guidelines, leaving workers protections at risk.

#### 3.2.2. Limited Availability of Personal Protective Equipment (PPE)

Further complicating the employer’s obligation to protect workers is the limited availability of personal protective equipment (PPE). Although the identification and classification of workers as “essential” can help local, state, and federal governments in the prioritization and allocation of resources, such as PPE, when resources are scarce, risks for essential workers may increase. For example, a critical shortage of surgical masks and N-95 respirators, which offer increased protection against COVID-19, has led to reserving these types of masks for healthcare workers who are most at risk [61]. However, scarcity has also led to permitting extended wear and re-use of these masks, thus raising concern about their effectiveness [62]. In addition, for non-healthcare essential workers, this critical shortage has meant that these workers must rely on other types of masks, such as cloth masks, for protection. Currently, the U.S. OSHA does not consider cloth face masks to be PPE [63] and therefore, employers are not required to provide cloth masks under the OSHA PPE standard. Unfortunately, this can increase the risk for moral injury as the burden of protection is placed on the employee who may be “ill-equipped” to select a cloth mask that offers adequate protection. 

#### 3.2.3. Lack of Sick Leave Polices and Recognition of COVID-19 as a Work-Related Disease

The lack of sick leave policies within the workplace may also result in increased risks to the worker. Although the details vary, many countries require paid sick leave to be provided to employees; however, the U.S. and the Republic of Korea are two economically developed countries that do not [64]. Data show that in the U.S. only half of workers in the lowest 25% of income have paid sick leave, while 92% of those in the top 25% of income have this benefit [65]. For those in the bottom 10% of income, the lack of coverage is even more dramatic as only 31% have access. This suggests that many essential “front-line” workers do not have paid sick leave. While the U.S. passed the Families First Coronavirus Response Act in March 2020 requiring certain employers to provide paid sick or family leave during the COVID-19 pandemic, data suggest that only a small percentage (~12%) of essential workers may be covered by this Act [66]. Without the added protection of paid sick leave, essential workers who are already economically disadvantaged are placed at even greater risk as they may lose their income or even their job to care for themselves or family members who fall ill [67]. As a result, workers exposed to COVID-19, whether they are experiencing symptoms or not, may feel as though they have no choice except to report to work [68].

For those who do fall ill following an exposure in the workplace, workers compensation programs can provide medical benefits and replacement of lost wages. In Brazil alone, data published in early June 2020 showed that over 16,000 worker compensation claims related to COVID-19 were in the process of being reviewed by the Labour Board [69]. However, given the structure of workers compensation in many countries, there is no guarantee that a worker who contracts COVID-19 would be covered [70]. The widespread nature of COVID-19, along with the lack of routine screening and testing in the workplace, can make it difficult to prove that a COVID-related illness or death is more likely than not due to a workplace exposure. 

In the early phase of the pandemic and as the number of workers’ compensation claims began to grow, several countries, including the U.S., Brazil, Mexico, and South Africa, began to critically evaluate their compensation claim approval process [69,70,71,72]. According to the National Conference of State Legislators, as of August 2020, only 14 states in the U.S. had taken action to include COVID-19 as a condition that can be covered by workers compensation [70]. Additionally, only 6 states were identified as having enacted presumption legislation that would help protect workers by placing the burden of proof on the employer to show that an infection was not work-related. Unfortunately, even if presumptive legislation exists, some state laws focus only on those at highest risk, such as healthcare staff and first responders, leaving workers in other essential industries without the benefit of added economic and health protections. 

In Canada, over 26,000 workers’ compensation claims have been filed related to COVID-19. Despite the difficulties in proving that COVID-19 was acquired in the workplace, the majority of claims (77%) have been accepted, which is encouraging [73]. However, even if a worker’s claim is accepted, employers are often incentivized, due to rising costs, to appeal the claim, and importantly, not all workers, including many essential workers, are even eligible to submit claims.

#### 3.2.4. Lack of Unemployment Benefits

For workers who are concerned about the lack of proper workplace protections, there is no law, at least in the U.S., that protects their job if they refuse to go to work due to COVID-related fears [74,75]. In addition, unemployment benefits, which vary from state to state, may not be readily available for those who leave their jobs due to fear of contracting COVID-19 unless they are identified as an at-risk population or able to demonstrate evidence of a workplace concern which often takes time and resources [74,75].

### 3.3. Recommended Actions for Protecting Essential Workers

Recognizing employment and working conditions as key factors that influence health, the World Health Organization (WHO) has promoted, within a justice context, actions to be taken at the global, national, and local level to provide fair employment and decent working conditions for all workers. Recommended actions include:

1. Making “full and fair employment and decent work a central goal of national and international social and economic policy-making;”
2. Providing “safe, secure, and fairly paid work, year-round work opportunities, and healthy work-life balance for all;” and
3. Improving “the working conditions for all workers to reduce their exposure to material hazards, work-related stress, and health-damaging behaviours” [76] (page 8).

Although providing only one aspect of a prevention scheme, the WHO specifically identifies employers as a having a key role in reducing hazards in the workplace and mitigating inequities that exist [76]. Therefore, emphasized below are actions that employers can take, even in the absence of strong local and federal regulations, to protect their workforce and help decrease the widening health inequity gap resulting from the COVID-19 pandemic. 

The implementation of a strong occupational health and safety program to identify and mitigate risks is one action that can help create a healthy workforce and reduce work-related illnesses and related harm [77]. The success of these programs is dependent on four elements: risk assessment of the workplace, hazard prevention and control, safety and health training, and management commitment with employee involvement [77]. Ideally, the foundation for an effective occupational health and safety program would be laid well before a time of crisis. However, even in their absence, all employers must take immediate action to protect their workforce and help control the spread of infection locally. In the context of COVID-19, this includes implementing key elements described by the U.S. CDC, as well as the WHO, such as [78,79]:

(1) Conducting frequent workplace assessments to identify risks and implementing mitigation strategies, using the hierarchy of controls, to protect all employees from physical and mental harm.

Employers should conduct workplace assessments, gathering information from a variety of sources, to identify COVID-19 risks. Walkthroughs of the facility should be conducted to examine all aspects of the work being performed and identify at-risk employees. Employees should be surveyed or interviewed to gain insight into potential risks and concerns. Existing policies and procedures should be closely examined. When identifying and implementing control strategies, employers should use the hierarchy of controls framework. This framework ranks control measures by their level of effectiveness, with elimination of a hazard being most effective, followed by engineering controls (e.g., modifying workstations to maintain proper distancing, installation of physical barriers, ensuring adequate ventilation, etc.), administrative controls (e.g., staggering work schedules, establishing disinfection procedures), and use of personal protective equipment (least effective). Employers should involve employees throughout the process and permit them to express concerns and discuss challenges they face during this current pandemic without fear of retribution. This may help employers better identify risks and implement actions that protect the workers’ physical as well as mental health. 

As more information is learned about the transmission of COVID-19 and the impact it has on working populations, employers must remain flexible. They must stay up-to-date on current recommendations offered by public health agencies, which continue to evolve over time, and implement mitigation strategies that offer the highest level of protection.

(2) Providing education and training for employees on how best to protect themselves and others.

Employers should share information from reliable public health sources with their employees regularly to ensure they have adequate knowledge to help protect themselves and others. This includes educating employees about potential risks and how to avoid them in a language that they can easily understand. Employers should provide them with training on how to work safely and properly use PPE. The education and training should also provide employees with additional information and resources needed to help recognize and overcome mental health stressors related to pandemic.

(3) Encouraging employees to report if they have been exposed or have infection.

Employers should encourage employees to report their potential exposures and if they have tested positive for COVID-19 infection. However, they should also have and enforce strict policies that protect workers from retribution for reporting such information. Employees must feel safe and trust that their employers will “do the right thing” when potential exposures and infections are reported. Rather than implementing punitive actions against the employee, employers should track and investigate any reported illnesses and exposures to identify areas in the workplace that require potential improvements and monitoring for the protection of other workers.

(4) Implementing policies that allow workers to stay home if they or a loved one is sick.

Employers must review their leave and pay polices and allow for greater flexibility to better support affected workers. Employees should not be in fear of losing their jobs if they need to take time off to care for themselves or a loved one. If changes are made to existing policies, the changes must be clearly explained to all employees. Employers should also strive to provide paid sick leave, even if there is no legal requirement that requires them to do so. 

## 4. Discussion

As the pandemic wears on and intensifies, it will be increasingly important to protect the essential workforce so that they can continue to work enabling critical infrastructure to remain operational. However, it is also important to protect these workers in their own right, from a social justice perspective. Evidence suggesting that many groups of essential workers are at increased risk of exposure to and infection from COVID-19 highlights inequities in social determinants of health that have been unfairly placed on this vulnerable population. These social determinants include the conditions (social, political, economic, and physical) in which people are born, live, work, play, and age that help shape one’s overall health status [76]. 

The provision of fair and decent work is essential for reducing existing social and health inequities [76]. Evidence has shown that adoption and enforcement of strong legislation and regulations can significantly improve overall working conditions and prevent occupational diseases [80]. Therefore, to protect both the physical and mental health of essential employees, specific pandemic-focused regulations requiring employers to implement the most protective precautions should be implemented. Additionally, to provide optimal protections, there should be: (1) requirements for employers to provide paid sick leave to all workers; (2) protections of unemployment benefits for workers who leave their job due to safety concerns; and (3) recognition of work-related infections in workers’ compensation claims with the burden of proof placed on employers to show that an infection was not work-related. Employers must also take immediate actions to implement worker protections. 

Employers have an ethical and moral responsibility to protect their workforce. Employers must recognize that workers in all critical industries are indeed essential and treat them as such by adopting a “duty of care” toward their health and safety. The duty-of-care ethic arises from a common law concept which argues that employers have an obligation to protect their employees against an unreasonable risk of harm [81]. Protection from harm includes not only preventing physical injury or illness, but also preventing occupational stressors that can negatively impact one’s mental health and overall well-being. 

While implementing many of the actions described above may be seen as an added cost for employers, significant benefits for the employer can be derived in both the short and the longer term. COVID-19 outbreaks can be avoided if an ill worker has sick leave and can stay home when ill, protecting other co-workers and avoiding large quarantines and plant shutdowns. Over the longer term, the implementation of paid sick leave policies has been shown to reduce job turnover [82], prevent workers from coming to work ill, reduce injuries, and increase access to preventive health services [83], making for a healthier workforce. One study, conducted pre-COVID, examined the economic impact of offering paid sick leave to reduce absenteeism related to an influenza-like illness and found significant monetary savings for employers [84].

Thus, although employers have an ethical and moral obligation to provide “reasonable care” toward their workers under common law requirements [85] and other regulations described above, there are benefits to be had for their enterprise by doing so. Proactively implementing optimal worker protections shows management commitment, which can help make employees feel that their health, safety, and well-being are valued. This can result in significant benefits for both the employee and the employer. Studies have shown that workers are more motivated and perform better overall if they trust their managers and employing organization [86]. It has also been shown that workers who felt valued and respected reported better overall well-being compared to those experiencing perceived injustice, lack of empathy or distrust [87]. Therefore, there is an evidence base for doing the right thing toward worker protection.

### Strengths/Limitations

This narrative review describes work-related COVID-19 risks and factors that increase these risks for essential workers not employed in the health sector, a population often overlooked at the start of the pandemic. One limitation of this review was the small number of scientific publications on this topic as the pandemic unfolded, requiring the use of available information in the gray literature and news sources. Inclusion of these additional sources may have introduced bias; however, every effort was made to include only references that cited information from “reliable” sources (e.g., national or local databases) and scientific literature, when available, was used to help support the information captured. In some cases, reports of illness and mortality among essential workers from the non-scientific literature did not contain denominators (total number of workers at risk), making it difficult to interpret the findings. The strength of this review is that it identifies common exposure scenarios that explain the dramatic differences in disease risk across multiple occupations and work sectors. While these observations are not novel, they demonstrate how basic, key occupational health and safety actions are disregarded by some employers, despite being recommended by prominent agencies, such as WHO and the CDC. Such actions described above suggest a way forward in the coming days of the pandemic, to better protect the lives and livelihoods of these essential workers.

## 5. Conclusions

The COVID-19 pandemic has increased not only work-related illness, but also moral injury of essential “front-line” workers across the world, illustrating additional examples of inequity and injustice for these vulnerable populations. While essential workers in the health sector are hailed as heroes, other essential workers are treated as “expendable”, with equivocal protections and uncertain legal recourse. Protection of both the physical and mental health of essential employees will require a multifaceted approach, including adoption and enforcement of strong occupational health and safety legislation and regulations, to address long-standing racial, ethnic, and gender inequities that exist. Employers must also assume the ethical and, in some jurisdictions, a legal obligation to adopt a duty of care toward their workers. Proactively implementing key elements of an occupational health and safety program in the workplace not only benefits workers by averting illness but promotes a safety climate that builds trust and resiliency in organizations. Workplace cultures modeled on safety may also prevent moral injury, further blunting the negative impact of COVID-19 on essential workers while bolstering their well-being, decreasing the health inequity gap, and stabilizing economic activity. 

## Figures and Tables

**Table 1:** Essential Worker Occupations *.

Category	Select Examples of Essential “frontline” Workers
Health Sector	Hospital workers Pharmacy workers Lab personnel Medical supplies and equipment providers Funeral home workers Long-term care workers Caregivers
Food and Agriculture Workers	Food processing/manufacturing workers Grocery and convenience store employees Farm workers Food service employees Warehouse workers
Law Enforcement/Public Safety/First responders	Firefighters Emergency medical service personnel Correctional officers Police officers Emergency service operators Security staff for buildings
Transportation and Logistics	Mass transit workers Trash collectors Postal and shipping employees Truck and bus drivers Warehouse employees Workers in the automotive industry
Essential Manufacturing	Workers involved in the manufacturing and supply of any essential equipment
Energy workers	Energy sector employees Petroleum/Natural gas sector employees Electric workers
Public Service workers	Public works employees Communication workers Plumbers/ electricians Road maintenance workers Building maintenance workers
Communications	Workers involvement in maintaining the communications infrastructure
Other essential workers	Workers that provide or support any necessary service (i.e., building supply workers, national security workers, financial service workers, etc.)

* Based on list provided by the U.S. Department of Homeland Security [11].

**Table 2:** Examples of Workplace COVID-19 Risks and Worker Impact.

Workers		Risk Factors Reported in Cited References	Reported Deaths/Reported Infections	Time Frame (Report Date)	Citation
**Food System Workers**	Meat and Poultry Processing	Difficulty physical distancing and poor hygiene Crowded living conditions and transportation Incentive pay to work may encourage workers to work while ill Lack of sick leave Cold, damp work environment Long work hours	20 deaths and 4913 reported infections in 130,578 workers in 115 processing plants across 19 U.S. states	8 May 2020	Dyal [27]
			≥48 deaths and ∼12,000 COVID-19 cases across two farms and 189 factories in the US	8 May 2020	Scher [28]
			Approximately 300 infections in one plant of 3700 workers in the US In one state, 38% of all infections (238 of 626) were workers in one meat packing company	14 April 2020	Rosane [29]
			2 deaths and 929 infected workers out of 3635 workers at one plant 210 (8.7%) of worker contacts also infected	7 August 2020	Steinberg [30]
			>1500 infections in German meatpackers 950 infections in Ireland meatpackers 20% of workers infected in one Dutch company 180 infections in two French slaughterhouses 10% of workers infected in one French slaughterhouse 200 infections in a Spanish meat plant	2 6 June 2020	Deutsche Welle [31]
			165 infections in one meatpacking plant in England	27 June 2020	Stewart [32]
			255 deaths and 50,123 infections in meatpackers across the U.S.	Data updated 1 December 2020	Douglas [33]
	Grocery store workers	Daily exposure to high volume of customers Inability to social distance Limited PPE * Politicization of masks leading to some customers refusing to wear Failure of government to enforce safety standards	68 deaths and > 10,000 infected in US	20 May 2020	Bradley [34]
			82 deaths and over 11,000 infected or exposed in first 100 days of pandemic in US	26 June 2020	Redman [35]
			21 of 104 (20%) workers infected in a single U.S. grocery store	Data collected in early May 2020	Lan [36]
	Grocery, retail, pharmacy, meatpacking, and other essential industries (UFCW ** members)	Weak safety standards	72 worker deaths and 5322 U.S. union workers “directly impacted” (tested positive for COVID-19, missed work due to self-quarantine, awaiting test results, or have been hospitalized, and/or are symptomatic)	11 September 2020	UFCW [37]
Law Enforcement/ Public Safety/First responders	Emergency responders	Interaction with public, including close contact during transport Administration of aerosol-generating treatments by emergency services personnel Suggested improper use of PPE	53 deaths in New York City emergency responders (fire and police)	20 May 2020	Guse [38]
			4 deaths and 5175 infections among 14,290 New York City firefighters and emergency services personnel (paramedics and emergency medical service technicians)	Based on data through 31 May 2020	Weiden [39]
	Officers and staff in correctional facilities	Lack of testing Lack of resources High rates of infection among prisoners (40% in one California prison)	Over 5000 infections among state and federal correctional officers	5 May 2020	Barr [40]
			6 deaths and 2169 infections in New York State	12 December 2020	New York State [41]
Transportation	Mass transit workers	Interaction with public Passengers not wearing masks Inability to social distance Limited availability of PPE and other safety supplies	120 deaths in New York City mass transit workers	20 May 2020	Guse [38]
			24% of approximately 3000 New York transit workers reported infection (compared to 19.9% in general population)	Data collected in August 2020	Gershon [42]
	Airline industry	Crowded working conditions Shortage of PPE (mask wearing reportedly banned initially) Non-notification of exposure to infected co-workers	15 U.S. deaths in nine days in April	20 April 2020	Feldman [43]
Factory workers		Cardboard barriers between worker stations COVID-19 guidance materials not translated into Spanish Lack of training on health protocols	4 deaths and 300 infections in one U.S. company with an estimated 2000 workers	20 July 2020	Friedman [44]
Doormen and women, janitors		Lack of PPE *	45 deaths in New York City	16 April 2020	Gould [45]

* PPE = personal protective equipment. ** UFCW = United Food and Commercial Workers International Union.

**Table 3:** Work-related Factors that May Increase Risk of Moral Injury in Essential Workers.

Conditions that May Increase Moral Injury Risk	Additional Details	Citations
Lack of Specific Federal Regulations or Standards	No federal OSHA standard or regulation outlines the precautions employers are required to control COVID-19 exposure OSHA’s General Duty Clause can be open to interpretation Per early CDC guidance, critical infrastructure workers may continue to work as long as asymptomatic and additional precautions taken	[57,59,60]
Limited availability of PPE	Reserving surgical masks and N95 respirators for healthcare workers Permitted extended wear and re-use of these masks Reliance on other types of masks, such as cloth masks, which may not be effective	[61,62,63]
Lack of Sick Leave Policies and Recognition of COVID-19 as a Work-related Infection	Lack of paid sick leave encourages workers to report to work Lack of routine workplace screening and testing leads to difficulty in proving that a COVID-related illness or death is from workplace exposure Lack of sufficient state legislation to include COVID as a condition to be covered by worker’s compensation Only 6 states enacted presumption legislation to help protect workers by placing the burden of proof on the employer	[64,65,66,67,68,69,70]
Lack of Unemployment Benefits	Unemployment benefits not available for those who leave jobs from fear of contracting COVID	[74,75]

## Data Availability

Data sharing not applicable.
